# Assessing the public health informatics workforce in Texas local health departments

**DOI:** 10.3389/fpubh.2026.1895129

**Published:** 2026-10-09

**Authors:** Rhoda A. Leos, Robert Hammarberg, Melissa Valerio-Shewmaker, Kimberly Baker

**Affiliations:** University of Texas Health Science Center at Houston (UTHealth Houston), School of Public Health, Houston, TX, United States

**Keywords:** health department informatics workforce, health department workforce needs, health informatics workforce, informatics workforce needs assessment, public health informatics

## Abstract

**Introduction:**

As health disparities persist and our technological capabilities continue to evolve, a well-trained and representative workforce in public health informatics has become crucial.

**Methods:**

A mixed-methods approach with purposive sampling was utilized to explore the current public health informatics infrastructure within Texas local health departments (Objective 1) and to identify current workforce assets and challenges (Objective 2). Data was collected via an online survey and virtual interviews with key informants. Survey questions included organizational-level questions relating to informatics-related practices and corresponded to specific maturity levels from the Capability Maturity Model Integration (CMMI) framework. Descriptive statistics and thematic analysis were utilized to summarize and synthesize insights derived from the data.

**Results:**

For questions regarding data collection, data sharing, and information dissemination, over half of local health departments (LHDs) selected responses at the “systematic” or “optimized” capability maturity levels (i.e., have department-wide processes supported by policies). However, almost half of LHDs selected responses in the “absent” or “initial” capability maturity levels (i.e., only *ad hoc* efforts exist) for questions regarding support for informatics-related training and support. Interviews further revealed that a wide range of staff and programs beyond epidemiology supported informatics-related practices; however, resource and personnel shortages posed challenges for the workforce.

**Discussion:**

Our findings highlight public health informatics as an applied practice integrated across various departmental programs in LHDs. Given multiple resource-related constraints, Texas LHDs face challenges in training and supporting their informatics workforce. These challenges may ultimately hinder capabilities to carry out informatics work at the optimized level.

## 1 Introduction

Health departments have faced many challenges in the integration and management of informatics within their organizations. While state health departments have contributed their expertise locally, local health department (LHD) staff must be able to carry out a wide range of informatics activities without the assistance of the state (1). A capacity and needs assessment survey conducted in 2015 described LHDs' top informatics activities as the extraction of data from information systems (69.5%) and the use and interpretation of quantitative (66.4%) and qualitative (55.1%) data while using geographic information systems (45.0%) or other analytical software (39.7%) (1). Unfortunately, research has shown that most public health professionals may not possess the necessary skills to use information effectively or even be aware of the importance of informatics (2). One study assessing awareness about public health trends found that only a little over half (55.6%) of LHD employees had any awareness that “leveraging electronic health information” was a public health trend (2). Furthermore, 49 in-depth key informant interviews with LHD professionals revealed that LHDs had too few informatics staff and limited informatics training (3). Other related concerns included “dated data, aging infrastructure, lack of training capacity, and insufficient time to complete tasks” (3).

These informatics-related challenges are also reflected in the Texas workforce. With a decentralized public health structure (local governments determine public health funding and services), Texas has over 170 local public health organizations (4, 5). Over 180 counties do not have an LHD and rely on the Texas Department of State Health Services (DSHS) for all public health services (6). According to the Association of State and Territorial Health Officials (ASTHO) Profile of State and Territorial Public Health, Texas DSHS had 36 health informatics specialist full-time equivalent positions (filled and vacant) in 2022, representing only 1% of the workforce (7). The Texas Tribune's database of compensation for Texas state employees also showed that 1% of DSHS employees were in a public health informatics specialist position as of July 2026 (8). While these percentages are not specific to local organizations or LHDs, past research has found that most LHDs lack a dedicated informatics team. According to NACCHO's most recent Public Health Informatics Profile, only 14% of LHDs reported having a dedicated informatics team or department in 2024, and those with a dedicated team were typically epidemiologists and IT staff (9). A 2025 study analyzing the most recent Public Health Workforce Interests and Needs Survey (PH WINS) data also found that public health informatics (PHI) specialists accounted for 0.5% of large LHDs (members of the Big Cities Health Coalition) and 0.3% of other LHDs (10). To date, no formal comprehensive needs assessment specific to the public health informatics workforce in Texas LHDs has been conducted.

Health informatics can be defined as “the science of how we collect, analyze, and use health information to improve health and healthcare” (11). It is a discipline that converges and shares methodologies with other fields such as data science and epidemiology (12). As a subfield, public health informatics can be defined as the “systematic application of information, computer science, and technology in areas of public health, including surveillance, prevention, preparedness, and health promotion” to “capture, manage, analyze, disseminate, and use information to improve population-level health outcomes” (13, 14). While many LHDs have tasked epidemiologists and IT staff to assist with informatics work, these traditional positions do not always reflect the specialized skills or distinct responsibilities of public health informatics professionals. Public health informatics professionals focus on “integrating and managing data, building stronger data systems, and improving how information flows across programs and partners” (15). The Applied Public Health Informatics Competency Model further outlines specific skill sets and abilities necessary to carry out public health informatics and address challenges in the field. These skills and abilities relate to eight competency areas: Analysis, Visualization, & Reporting; Communications; Evaluation; Information Systems; Policy; Principles & Strategies; Project Management; and Standards & Interoperability (16). These public health informatics competencies ultimately affect LHDs' ability to address health disparities. Past research examining the effect of public health informatics in addressing disparities found that LHDs with greater use of information systems had “higher levels of engagement in performing activities to address health disparities and health inequities” (17).

With a vision to address the challenges outlined in the literature and expand efforts that would strengthen the public health informatics workforce across Texas, the University of Texas Health Science Center at Houston (UTHealth Houston) launched the Gaining Equity in Training for Public Health Informatics and Technology (GET PHIT) initiative and statewide consortium in 2021. The consortium was established with nine partnering Texas universities, which would drive the initiative statewide. With funding from the Assistant Secretary for Technology Policy (ASTP) within the U.S. Department of Health and Human Services (HHS), the consortium used a comprehensive approach that included bootcamps and internships for university students, professional development courses for workforce professionals, and a workforce needs assessment. The workforce needs assessment was a critical and necessary piece of the initiative that would explore informatics capacities and gaps specific to LHDs and LHD staff in Texas.

## 2 Methods

### 2.1 Study design and tools

A mixed-methods approach was utilized to conduct an exploratory study to gain insight into the current public health informatics workforce infrastructure within Texas LHDs (objective 1) and to identify key assets and challenges (objective 2). Literature and assessment tools were reviewed to guide the design of the study's data collection tools. With feedback from GET PHIT consortium members, a participant survey and interview guide were finalized to include participant demographic questions (e.g., age, gender, education) and organizational-level questions adapted from existing self-assessment tools published by the Public Health Informatics Institute and the Kansas Health Institute.

Organizational-level questions aimed to investigate several informatics-related areas, including data collection, data sharing processes, and informatics training. Sample questions included: “Do you currently have procedures for establishing data sharing agreements?”, “Does your organization use audience-specific messaging to communicate the value of public health data in your community?” and “Does your organization support staff members across a broad range of job classifications to participate in informatics training?” Each response option corresponded to a specific maturity level from the Capability Maturity Model Integration (CMMI) framework, designed to evaluate organizational capabilities and offer guidance for continuous improvement (18). We used two versions of the scale to mirror the Building an Informatics-savvy Health Department: A Self-Assessment Tool and the Kansas Public Health Informatics Self-Assessment Tool (19, 20). Table 1 defines the maturity levels for each version.

**Table 1:** Capability Maturity Model Integration (CMMI) framework – maturity scales.

Response option	Corresponding maturity level	Maturity level definition
Version 1		
Source: building an informatics-savvy health department: a self-assessment tool^*^		
0	Absent	No capability is evident; “starting from scratch.”
1	Initial	No organized, systematic efforts to build informatics capacity exist, only *ad hoc* efforts and isolated, individual heroics.
2	Managed	Some organized efforts have begun or been completed but are not systematically documented or institutionalized.
3	Defined	Systematic, ongoing efforts are underway, but there are no overall method to measure progress or to ensure coordination.
4	Measured	Systematic, ongoing efforts are underway to measure progress and ensure coordination.
5	Optimized	Systematic, ongoing efforts are underway with quality improvement activities to align results with guiding vision, strategies, and performance metrics.
Version 2		
Source: Kansas public health informatics self-assessment tool^**^		
0	Absent	No capability is evident.
1	Sporadic	Some organized efforts in one or more programs of agency, but no methods to assess need or improve performance.
2	Systematic	Agency-wide processes are supported by agency policies, but with no method to assess needs or improve performance.
3	Optimized	Agency-wide processes are supported by agency policies and include methods to assess needs and improve performance.

^*^Survey questions adapted from the Informatics-Savvy Health Department tool used version 1 (v1).

^**^Survey questions adapted from the Kansas Public Health Informatics tool used version 2 (v2).

### 2.2 Recruitment and data collection

Partnerships between UTHealth Houston, academic institutions, and public health agencies were established before the initiative's official launch. Through these partnerships, purposive sampling was used to identify and recruit LHD staff with insight into their organization's informatics-related work. Beginning in April 2022, recruited LHD staff were sent a link with the study's information letter and survey. Once a participant submitted their survey, they were contacted by the research team to schedule a 1-h virtual interview. Each interview was transcribed via WebEx and reviewed for accuracy. Interviews and data collection concluded in September 2022.

### 2.3 Data analysis

Survey data were reviewed for missingness and uploaded into Stata for analysis. Descriptive statistics summarized demographics, including participants' years of experience, years employed by the organization, gender, race, ethnicity, education, and age. Responses to informatics-related questions were summarized according to three activity categories: data collection, data sharing, and dissemination of information; informatics-related training, support, and resources; and information partnerships to meet community needs. Capability maturity levels were summarized with counts and percentages.

Interview data were analyzed using a multi-step process to identify key themes and patterns. First, a research team member reviewed the recordings and transcripts to ensure the accuracy of the transcripts. After transcripts were finalized, sentences and paragraphs were organized into meaningful segments and assigned labels or “codes” that aligned with the study's objectives. One researcher conducted this initial coding process, while a second researcher reviewed the coded data, specifically looking for potential gaps or discrepancies. Both researchers met several times to discuss differences in interpretation and to finalize codes. After finalizing the codes, emerging patterns or themes were identified and finalized through the same process (one researcher doing the initial round and the second researcher reviewing before meeting together). Themes were presented to the larger team before finalizing to ensure clarity. Finally, a narrative was written to present each theme, supported by examples and direct quotes.

## 3 Results

### 3.1 Demographics

After contacting 12 partner LHDs, a total of 11 LHDs responded and helped us identify one staff member to participate on behalf of their department. The 11 staff members who participated included a Data Services Program Manager, Epidemiology and Surveillance Program Manager, Data Analytics Reporting Manager, Informatics Manager, Chief Epidemiologist, Chief of Informatics, Chief of Staff, Assistant Health Administrator, two Directors, and an Assistant Director. Most participants identified as female (*n* = 10), White (*n* = 8), and non-Hispanic/Latino (*n* = 8). Additionally, most were 35+ years of age (*n* = 9) and held a master's degree (*n* = 8). The number of years employed by their department ranged from 1–3 years to 10 years+, with the highest percentage being employed for 10 years+ (36.4%). Years of professional public health and informatics experience ranged from no experience to 10 years+. Participants had more years of professional experience in public health (63.6% had a minimum of 7 years) than informatics-specific experience (27.2% had a minimum of 7 years). A complete summary of participant demographics is provided in Table 2. Most participating LHDs (*n* = 10) were classified as “large” by the Texas Association of City and County Health Officials (TACCHO). Almost half of the participants (*n* = 5) reported their departments having 100–500 employees, with over half (*n* = 6) noting that ethnic minorities comprised over 75% of the workforce. While most lacked a dedicated informatics unit, they confirmed having IT support for informatics projects. Table 3 provides a demographic overview of each represented health department.

**Table 2:** Participant demographics (*N* = 11).

Characteristics	Category	*n* (%)
Gender	Female	10 (90.9)
	Male	1 (9.1)
Age	24–34	2 (18.2)
	35–44	5 (45.5)
	45–54	1 (9.1)
	55+	3 (27.3)
Ethnicity	Hispanic or Latino	3 (27.3)
	Not Hispanic or Latino	8 (72.7)
Race	Asian	2 (18.2)
	Multiracial	1 (9.1)
	White	8 (72.7)
Highest Level of Education	Bachelor's degree	1 (9.1)
	Master's degree	8 (72.7)
	Doctoral or professional	2 (18.2)
	1–3 years	2 (18.2)
Years of professional experience in public health	4–6 years	2 (18.2)
	7–9 years	2 (18.2)
	10+ years	5 (45.5)
	No experience	1 (9.1)
Years of professional experience in public health informatics	Less than 1 year	3 (27.3)
	1–3 years	2 (18.2)
	4–6 years	2 (18.2)
	7–9 years	1 (9.1)
	10+ years	2 (18.2)
	1–3 years	3 (27.3)
Years employed by organization	4–6 years	2 (18.2)
	7–9 years	2 (18.2)
	10+ years	4 (36.4)
	Less than 1 year	2 (18.2)
Years in current position	1–3 years	5 (45.5)
	4–6 years	1 (9.1)
	7–9 years	2 (18.2)
	10+ years	1 (9.1)
	25–34	2 (18.2)
Age	35–44	5 (45.5)
	45–54	1 (9.1)
	55+	3 (27.3)

**Table 3:** Health department demographics.

ID	Type^*^	Size^*^	Public health region	Employees^*^		Informatics unit/chief informatics officer^**^
				Number	% Ethnic minorities	
01	City	Large 1	9/10	100–500	>75%	No/In progress
02	City	Large 2	6/5	1,001–5,000	>75%	Yes
03	City	Medium	11	100–500	>75%	No/In progress
04	District	Large 2	8	501–1,000	51%−75%	Yes
05	City	Large 1	1	100–500	26%−50%	No/In progress
06	County	Large 1	11	100–500	>75%	No/In progress
07	County	Large 2	6/5	5,001–10,000	26%−50%	No/In progress
08	County	Large 2	2/3	*No response*	*No response*	Yes
09	County	Large 2	2/3	501–1,000	>75%	No/In progress
10	City/County	Large 2	7	501–1,000	51%−75%	No/In progress
11	City	Large 1	11	100–500	>75%	No/In progress

^*^Based on TACCHO classification (Small = population up to 49,00; Medium = population 50,000 – 249,999; Large Category 1 = population 250,000–999,999; Large Category 2 = population 1 million+).

^**^Based on self-reported data.

### 3.2 Survey - maturity levels

When asked about data collection, sharing, and information dissemination processes, over 50% of LHDs selected responses at the systematic level (where department-wide processes are supported by policies) or at the optimized capability maturity level (where department-wide processes are supported by policies and methods to assess needs and improve performance exist). Regarding informatics training, resources, and support, most LHDs (90%) indicated that informatics training was not part of their organizational plan. When asked about supporting staff across various job classifications to participate in informatics training, almost half (45.5%) of LHDs selected responses at the absent or initial capability maturity levels (where there is no capability or only *ad hoc* efforts). When asked about having appropriate job classifications for informatics positions, over 60% selected responses at either the absent or initial maturity levels. Regarding information partnerships, almost fifty percent indicated they “sometimes” invite community leaders from marginalized groups to participate in public health data system design, content, or use discussions. Additionally, 27% of LHDs selected responses at the optimized maturity level when asked about collaborating effectively with community partners for population health assessment. Results for all survey questions are provided in Table 4.

**Table 4:** Survey results & capability maturity levels.

Question	CMMI scale version^*^	Response options	Maturity level	*n* (%)
I. Data collection, data sharing, and dissemination of information				
Do you currently have procedures regarding the collection and documentation of public health data?	v2	0-We have no such procedures for collection and documentation of data.	Absent	0 (0)
		1-We have procedures for collection and documentation of data within some programs.	Sporadic	4 (36.4)
		2-We have documented procedures for collection and documentation of data but have no method to assess needs or improve performance.	Systematic	2 (18.2)
		3-We have documented procedures for collection and documentation of data and have methods to assess needs and improve performance.	Optimized	5 (45.5)
Do you currently have procedures for establishing data-sharing agreements?	v2	0-We have not adopted such a procedure.	Absent	0 (0)
		1-We have some data-sharing agreements but have no procedures governing their development.	Sporadic	2 (18.2)
		2-We have procedures for establishing data-sharing agreements but have no method to assess needs or improve performance.	Systematic	5 (45.5)
		3- Agency has procedures for establishing data-sharing agreements and has methods to assess needs and improve performance.	Optimized	4 (36.4)
Do you have processes in place to disseminate summary reports or other pertinent information?	v2	0-We have no such processes to disseminate summary reports and other pertinent information.	Absent	0 (0)
		1-We allow programs to disseminate summary reports and other pertinent information by their own processes.	Sporadic	1 (9.1)
		2-We have processes in place for the dissemination of summary reports and other pertinent information but have no method to assess needs or improve performance.	Systematic	5 (45.5)
		3-We have processes in place for the dissemination of summary reports and other pertinent information and have methods to assess needs and improve performance.	Optimized	5 (45.5)
Does your department or organization have a communication system to electronically alert stakeholders of new or ongoing emergencies on a 24/7 basis?	v2	0-Agency has no system for electronic notification.	Absent	2 (18.2)
		1-Agency has an electronic notification system to electronically alert some stakeholders.	Sporadic	2 (18.2)
		2-Agency has a communication system to electronically alert stakeholders with defined procedures for its use but has no method to assess needs or improve performance.	Systematic	5 (45.5)
		3-Agency has a communication system to electronically alert stakeholders with defined procedures for its use and has methods to assess needs and improve performance.	Optimized	2 (18.2)
Does your organization use audience-specific messaging to communicate the value of public health data in your community?		Rarely		1 (9.1)
		Sometimes		3 (27.3)
		Often		3 (27.3)
		Always		3 (27.3)
		No response		1 (9.1)
II. Informatics-related training, support, and resources				
Has your department/organization conducted an inventory of its data sets, applications, and information systems?	v1	0-We have not conducted an inventory of data sets, applications, and information systems.	Absent	1 (9.1)
		1-We have made isolated, *ad hoc* efforts to inventory our data sets, applications, and information systems.	Initial	4 (36.4)
		2-We make regular attempts to conduct inventories of our data sets, applications, and information systems.	Managed	5 (45.5)
		3-We have established a process for routine inventories but do not evaluate the results.	Defined	0 (0)
		4-We routinely evaluate the findings following inventories of our data sets, applications, and information systems.	Measured	1 (9.1)
		5-We use findings from inventories to improve the availability of information to meet health department goals.	Optimized	0 (0)
Does your department/organization support staff members across a broad range of job classifications to participate in informatics training?	v1	0-We provide no support for staff members to participate in public health informatics training.	Absent	3 (27.3)
		1-Policies and practices exist that support staff members to receive informatics training; however, they are not known, are applied unevenly, or are rarely used.	Initial	2 (18.2)
		2-Policies and practices exist to support staff to participate in informatics training for a limited set of staff positions.	Managed	5 (45.5)
		3-Policies and practices exist that support informatics training for a broad range of job classifications.	Defined	1 (9.1)
		4-We evaluate the informatics training opportunities made available to staff members.	Measured	0 (0)
		5-We use evaluation findings to improve informatics training opportunities for staff members.	Optimized	0 (0)
Does your department/organization have appropriate job classifications, including position descriptions and pay scales for informatics positions?	v1	0-We have not attempted to adopt such informatics job classifications.	Absent	3 (27.3)
		1-We have made initial, isolated *ad hoc* efforts to adopt such informatics job classifications.	Initial	4 (36.4)
		2-We have made sustained attempts to adopt such informatics job classifications.	Managed	0 (0)
		3-We have adopted such informatics job classifications and established a method to evaluate implementation.	Defined	1 (9.1)
		4-We have adopted such informatics job classifications and established a method to evaluate implementation.	Measured	1 (9.1)
		5-We use evaluation findings to improve how it recruits and retains informatics professionals.	Optimized	1 (9.1)
		No response	n/a	1 (9.1)
Do you have some form of informatics training included in an organizational plan (e.g., strategic plan, workforce development plan, etc.)		No		10 (90.9)
		Yes		1 (9.1)
		Does your department or organization have equipment or software needed to support informatics-related projects?		Some (but not sufficient)		5 (45.5)
		Yes		6 (54.6)
Are there funds currently earmarked for the support of informatics-related projects?		No		5 (45. 5)
		Yes		6 (54.6)
iii. Information partnerships to meet community needs				
Does your department/organization have the capability to conduct a comprehensive community health assessment and identify health priorities from that assessment?	v2	0-We have no capability to conduct a community health assessment.	Absent	1 (9.1)
		1-We use some tools and resources when conducting a community health assessment, but it is not comprehensive.	Sporadic	1 (9.1)
		2-We use data and tools/resources when conducting a community health assessment and prioritizing health issues but have no method to assess needs or improve performance.	Systematic	2 (18.2)
		3-We use data and tools/resources when conducting a community health assessment and prioritizing health issues and have methods to assess needs and improve performance.	Optimized	6 (54.6)
		No response	n/a	1 (9.1)
Does the department/organization effectively collaborate with community partners to use information for population health assessment and improvement?	v1	0-We do not collaborate in using information in this way.	Absent	0 (0)
		1-We make rare, *ad hoc* efforts to collaboratively use information for population health assessment and improvement.	Initial	1 (9.1)
		2-We collaborate but not systematically or in an ongoing way in using information for population health improvement.	Managed	2 (18.2)
		3-We have ongoing established collaborative relationships to use information for population health improvement.	Defined	1 (9.1)
		4-We have established collaborative relationships with such partners and evaluate how well these collaborations support population health improvement.	Measured	3 (27.3)
		5-We use evaluation findings to improve our collective use of information to assess and improve population health.	Optimized	3 (27.3)
		No response	n/a	1 (9.1)
Are community leaders or representatives from marginalized groups invited to participate in discussions around public health data system design, content, and/or use?		Always		2 (18.2)
		Often		1 (9.1)
		Sometimes		5 (45.5)
		Rarely		1 (9.1)
		Never		1 (9.1)
		No response		1 (9.1)

^*^Refer to Table 2 CMMI scale versions.

### 3.3 Interviews

While we aimed to interview the same participants who completed the survey, four participants were no longer available due to retirement, leave, or limited availability. Three of those participants referred us to other staff who could participate on their behalf. Two participants also invited additional team members to be present in the interview. With these changes, we completed a total of 10 interviews (representing 10 of the initial 11 LHDs) with 14 individuals (two interviews had more than one representative). After transcribing and analyzing interview data, we identified three key themes from the qualitative data that highlight the public health informatics infrastructure and the challenges and assets of the workforce in local health departments. While epidemiology teams were often considered central to the informatics infrastructure, other internal programs supported informatics practices (Theme 1). Personnel and resource constraints emerged as significant challenges impacting the ability to conduct informatics work and training (Theme 2). Additionally, established partnerships, both internal and external, proved to be crucial for successful informatics efforts (Theme 3). These themes will be explored further in the following sections.

#### 3.3.1 Theme 1 – multiple units supporting public health informatics

Participants explained how informatics is used in their departments, often referring to “Epidemiology,” “Epidemiologist,” and “Epi staff” for those at the forefront of informatics-related work. Some departments had only one or a few epidemiologists, while others had larger units or sub-teams. An Assistant Director mentioned that their department relies on a single epidemiologist responsible for collecting and organizing data from environmental and clinical sites. While “epidemiology” and “informatics” were often used interchangeably, it was evident that other programs contributed to informatics-related work. One Assistant Director stated, “We are a department that provides direct services…in every division, even though it's segmented, there's some sort of tracking mechanism that monitors the information and productivity through data.” Both clinical and non-clinical programming, such as STD/HIV, tuberculosis, health promotion, and environmental health programs, were identified as actively collecting and sharing data via an electronic medical record (EMR) system or other data reporting systems. Other examples included a workforce development program consisting of a data management team and an information technology team overseeing data access and storage. Participants mostly discussed these programs' role in data sharing, collection, and management, rather than their role in a formal informatics infrastructure.

#### 3.3.2 Theme 2 – resource-related constraints impacting the workforce

As participants discussed workforce gaps or challenges, it was evident that funding and retaining staff were key constraints impacting the ability to pursue informatics-related work efficiently. Although COVID grants made it possible to hire some additional staff to meet pandemic-related demands, participants expressed concerns about funds coming to end and not being able to retain those staff. Past hiring experiences were also shared, and the difficulty in finding professionals with sufficient informatics-related expertise and skills beyond a general master's degree in public health was a challenge. Some participants faced staff turnover and loss of employees to higher-paying opportunities. The shortage in staff was also attributed to underdeveloped training efforts. Participants felt it would be difficult to expand training without the proper staff to lead or oversee it.

Another key constraint was specific to the software and reporting systems used for data collection and sharing. Either the systems were outdated or inoperable. One participant expressed difficulties utilizing a state reporting system and explained, “That's why we had all these cities and municipalities create their systems on the fly for COVID, because the system that the state had cannot handle it?.” Another stressed the difficulties of using internal reporting systems that do not communicate. The participant further explained, “We maintain different systems within our EPI, within our TB or TIs, and then the notification to the state. That's a completely different system, even between the different programs. So, we're talking about six different systems that…don't communicate with each other.?” Some noted they were modernizing software and reporting systems and hiring staff. Others lacked the funding to modernize in the ways they desired.

#### 3.3.3 Theme 3 – partnerships as an asset and strategy for informatics work

All departments faced personnel and resource challenges in informatics work, but they viewed partnerships as a key strategy to mitigate such challenges. Partnerships (formal and informal) with other departments and local government agencies were highlighted by most participating LHDs. One participant stated, “we recently partnered with our department of Human Services Department to collect data on youth mental health, that's been a priority area for us because mental health is incredibly fragile right now for everybody.” Participants shared other examples of partnerships and collaborations, such as working with the IT department on database upgrades, partnering with the Fire Department for COVID data management, and collaborating with the GIS Department to create interactive dashboards. External partnerships were also emphasized, involving stakeholders like universities, non-profits, hospital providers, school districts, and Texas DSHS. One participant expressed, “It wasn't hard to get data from the hospital providers… we have some automatic feeds to get all of their COVID testing and vaccine data.” Another described their department's involvement in a community partners group where multiple stakeholders gather to share data. Some participants mentioned having formal contracts with stakeholders, including one who highlighted their department's agreement with an academic institution for PhD students to assist with data analysis. These collaborations illustrate the entities sharing common goals in public health informatics and the importance of trust and lasting relationships.

## 4 Discussion

Our findings highlight public health informatics as an applied practice woven across multiple department programs and divisions, rather than the responsibility of a single unit. While epidemiologists are often regarded as informatics leads in LHDs, our assessment and exploration of the local workforce in Texas reveals a wider range of staff and programs becoming involved in day-to-day informatics-related practices. As more diverse staff take on informatics work, it is important to modernize job classifications to better define roles and avoid skill gaps. The constraints emphasized by participating LHDs reaffirm what other LHDs across the U.S. have faced and the need to expand resources and training (beyond Epi staff or a single program). While the COVID-19 pandemic added unprecedented burden to the public health workforce as a whole, LHDs were specifically impacted by overwhelming data-related demands, pressuring already limited informatics resources and staff. Nonetheless, our findings underscore how informatics partnerships have served as assets and helped mitigate resource gaps. It is through collaboration and partnership development that many informatics-related efforts and initiatives have been maximized during and post-COVID.

Given the limited sample size in this study, we cannot generalize findings to all Texas LHDs. Most LHDs in our sample were large health departments (according to TACCHO size definitions), which typically have more robust infrastructure and resources compared to smaller or rural LHDs. Differences also exist between LHDs and state agencies; therefore, we cannot generalize findings to the Texas DSHS informatics workforce. A future workforce needs assessment comparing local- and state-level challenges could provide further insight. Additionally, the terms “informatics” and “public health informatics” are broad terms that encompass a wide range of competencies and activities. While we attempted to recruit staff with sufficient knowledge of these terms and their application, we did not formally assess participants' knowledge or expertise in public health informatics. We also categorized informatics activities very broadly and did not compare them by organization level (e.g., operational vs. strategic) or by competency category (e.g., principles & strategies vs. information systems). Future research would benefit from these considerations to better identify areas of improvement. Regarding our interview findings, we acknowledge the possibility of participation/non-response bias due to our sampling strategy, as well as interviewer/interviewee bias. While we discuss partnerships as a key theme in our findings, we do not formally assess the quantity or quality of informatics partnerships across participating departments. Given the contributions of partner organizations, high and low maturity levels may be a reflection of the quantity and quality of those partnerships vs. workforce capacity and skills alone. This is another consideration for future research.

## Data Availability

The original contributions presented in the study are included in the article/Supplementary material, further inquiries can be directed to the corresponding author.
