# Supplementary material for: Assessing the public health informatics workforce in Texas local health departments
Source: Front Public Health. 2026 Oct 9;14:1895129. doi: 10.3389/fpubh.2026.1895129 (PMC13690701; doi:10.3389/fpubh.2026.1895129)
Supplement: Supplementary file 1 [file Data_Sheet_1.pdf]

Qualtrics Survey

Thank you for your interest in taking part in this study and completing the following survey. Your responses will help us identify current needs relating to the public health informatics infrastructure and workforce in Texas. The Centers for Disease Control and Prevention (CDC) defines public health informatics as the systematic application of information, computer science, and technology to public health practice, research, and learning. The application of informatics in public health includes the process by which raw data turns into digestible information to enable effective monitoring and surveillance and ensuring that practitioners have access to timely and accurate data. (https://phii.org/how-we-do-it/defining-public-health-informatics/)

The information collected in this survey will not be shared with anyone and will be reported in the aggregate form only.

I. About You

To begin, please provide your background information.

Department/organization name:	
Current position title:	
Years of professional experience in public health	❑ No experience<br>❑ Less than one year<br>❑ 1-3 years<br>❑ 4-6 years<br>❑ 7-9 years<br>❑ 10+ years
Years of professional experience in public health informatics	❑ No experience<br>❑ Less than one year<br>❑ 1-3 years<br>❑ 4-6 years<br>❑ 7-9 years<br>❑ 10+ years
Do you possess any certifications?	❑ Yes (If yes, please state certification title and awarding institution):<br>____________________________________<br>❑ No
Number of years in your organization	❑ Less than one year<br>❑ 1-3 years<br>❑ 4-6 years<br>❑ 7-9 years<br>❑ 10+ years
Number of years in your current position	❑ Less than one year<br>❑ 1-3 years<br>❑ 4-6 years<br>❑ 7-9 years<br>❑ 10+ years
Gender	❑ Male

	❑ Female<br>❑ Transgender<br>❑ None of these
Age (years)	❑ 18-24<br>❑ 25-34<br>❑ 35-44<br>❑ 45-54<br>❑ 55+
Ethnicity	❑ Hispanic or Latino<br>❑ Not Hispanic or Latino
Race	❑ White<br>❑ Black or African American<br>❑ American Indian or Alaska Native<br>❑ Asian<br>❑ Native Hawaiian or Other Pacific Islander<br>❑ Multiracial<br>❑ Other<br>❑ Unknown/Unsure
Highest level of education you have completed	❑ Some High School<br>❑ High School<br>❑ Bachelor's Degree<br>Area of study: _________________<br>❑ Master's Degree<br>Area of study: _________________<br>❑ Doctoral or professional<br>Area of study: _________________<br>❑ Vocational/technical school<br>Area of study: _________________

II. About the Public Health Department/Organization

Please answer the following questions to the best of your knowledge.

How many employees are currently in your department or organization?	❑ <100<br>❑ 100-500<br>❑ 500-1,000<br>❑ 1,000-5,000<br>❑ 5,000-10,000<br>❑ 10,000+
What percentage of your employees are considered ethnic minorities?	❑ Under 25%<br>❑ 26 - 50%<br>❑ 51-75%<br>❑ Over 75%
Do you currently have an informatics unit or Chief Informatics Officer with cross-agency responsibility and authorities, including those	❑ Yes<br>❑ No

related to the health department's information vision, strategies and polices?	If yes, please list key responsibilities or duties: _________________________
What program or department is your informatics unit/officer housed under?	❑ IT (Information Technology)<br>❑ Epidemiology<br>❑ Other: _________________________
Is there IT support for informatics projects and goals?<br><br>*(IT refers to a team or department in an organization that provides technical help to people who have problems with its computer systems)*	❑ Never<br>❑ Rarely<br>❑ Sometimes<br>❑ Often<br>❑ Always
Do you currently have procedures regarding the collection and documentation of public health data? (If more than one is true, please select the option with the highest number)	❑ 0 - We have no such procedures for collection and documentation of data.<br>❑ 1 - We have procedures for collection and documentation of data within some programs.<br>❑ 2 - We have documented procedures for collection and documentation of data but have no method to assess needs or improve performance.<br>❑ 3 - We have documented procedures for collection and documentation of data and have methods to assess needs and improve performance.
Do you currently have procedures for establishing data-sharing agreements? (If more than one is true, please select the option with the highest number)	❑ 0 - We have not adopted such a procedure.<br>❑ 1 - We have some data-sharing agreements but have no procedures governing their development.<br>❑ 2 - We have procedures for establishing data-sharing agreements but have no method to assess needs or improve performance.<br>❑ 3 - Agency has procedures for establishing data-sharing agreements and has methods to assess needs and improve performance.
Do you have processes in place to disseminate summary reports or other pertinent information? (press releases, issue briefs, regular epidemiology updates, etc.) (If more than one is true, please select option with highest number)	❑ 0 - We have no such processes to disseminate summary reports and other pertinent information.<br>❑ 1 - We allow programs to disseminate summary reports and other pertinent information by their own processes.<br>❑ 2 - We have processes in place for the dissemination of summary reports and other pertinent information but have no method to assess needs or improve performance.

	❑ 3 - We have processes in place for the dissemination of summary reports and other pertinent information and have methods to assess needs and improve performance.
Has your department/organization conducted an inventory of its data sets, applications and information systems? (If more than one is true, please select the option with the highest number)	❑ 0 - We have not conducted an inventory of data sets, applications and information systems.<br>❑ 1 - We have made isolated, ad hoc efforts to inventory our data sets, applications and information systems.<br>❑ 2 - We make regular attempts to conduct inventories of our data sets, applications and information systems.<br>❑ 3 - We have established a process for routine inventories but do not evaluate the results.<br>❑ 4 - We routinely evaluate the findings following inventories of our data sets, applications and information systems.<br>❑ 5- We use findings from inventories to improve the availability of information to meet health department goals.
Does your department/organization have a communication system to electronically alert stakeholders of new or ongoing emergencies on a 24/7 basis? (If more than one is true, please select the option with the highest number)	❑ 0 - Agency has no system for electronic notification.<br>❑ 1 - Agency has an electronic notification system to electronically alert some stakeholders.<br>❑ 2 - Agency has a communication system to electronically alert stakeholders with defined procedures for its use but has no method to assess needs or improve performance.<br>❑ 3 - Agency has a communication system to electronically alert stakeholders with defined procedures for its use and has methods to assess needs and improve performance
Do you have some form of informatics training included in an organizational plan (e.g. strategic plan, workforce development plan, etc.)<br><br>If yes, would you be willing to share that plan/document with us?	❑ Yes<br>❑ No<br><br>❑ Yes<br>❑ No
Does your department/organization support staff members across a broad range of job classifications to participate in informatics training? (If more than one is true, please select the option with the highest number)	❑ 0 - We provide no support for staff members to participate in public health informatics training.<br>❑ 1 - Policies and practices exist that support staff members to receive informatics training; however, they are not known, are applied unevenly or are rarely used.

	❑ 2 - Policies and practices exist to support staff to participate in informatics training for a limited set of staff positions.<br>❑ 3 - Policies and practices exist that support informatics training for a broad range of job classifications.<br>❑ 4 - We evaluate the informatics training opportunities made available to staff members.<br>❑ 5 - We use evaluation findings to improve informatics training opportunities for staff members.
Which specific training topics would your current staff benefit from the most? Please select the top 5.<br><br>*(See table below for topic descriptions.)*	❑ Intro to Public Health Informatics<br>❑ Epidemiology & Public Health Informatics<br>❑ Health Data Science<br>❑ Privacy and Security<br>❑ Health Equity<br>❑ Public Health Analytics<br>❑ Surveillance in Public Health<br>❑ Public Health Reporting<br>❑ Semantic Interoperability<br>❑ Public Policy<br>❑ Multi-stream Data Management<br>❑ Social Media Listening<br>❑ Health Literacy<br>❑ Racism & Bias in Data Use<br>❑ Bias in Machine Learning and Artificial Intelligence
Does your department/organization have equipment or software needed to support informatics-related projects?	❑ Yes<br>❑ No<br>❑ Some, but not sufficient
Are there funds currently earmarked for the support of informatics-related projects?	❑ Yes<br>❑ No<br>If yes, how are these funds obtained?<br><br>_________________________________
Does your department/organization have the capability to conduct a comprehensive community health assessment and identify health priorities from that assessment? (If more than one is true, please select the option with the highest number)	❑ 0 - We have no capability to conduct a community health assessment.<br>❑ 1 - We use some tools and resources when conducting a community health assessment, but it is not comprehensive.<br>❑ 2 - We use data and tools/resources when conducting a community health assessment and prioritizing health issues but have no method to assess needs or improve performance.<br>❑ 3 - We use data and tools/resources when conducting a community health assessment

	and prioritizing health issues and have methods to assess needs and improve performance.
Does the department/organization effectively collaborate with community partners for population health assessment and improvement? (If more than one is true, please select the option with the highest number)	❑ 0 - We do not collaborate in using information in this way.<br>❑ 1 - We make rare, ad hoc efforts to collaboratively use information for population health assessment and improvement.<br>❑ 2 - We collaborate but not systematically or in an ongoing way in using information for population health improvement.<br>❑ 3 - We have ongoing established collaborative relationships to use information for population health improvement.<br>❑ 4 - We have established collaborative relationships with such partners and evaluate how well these collaborations support population health improvement.<br>❑ 5 - We use evaluation findings to improve our collective use of information to assess and improve population health.
Are community leaders/representatives from marginalized groups invited to participate in discussions around public health data system design, content, and/or use?	❑ Never<br>❑ Rarely<br>❑ Sometimes<br>❑ Often<br>❑ Always
Does your department/organization use audience-specific messaging to communicate the value of public health data in your community?<br><br>*(Audience-specific messaging refers to information that has been tailored to a specific group's values, needs, and/or experiences for the purpose of better connecting with them.)*	❑ Never<br>❑ Rarely<br>❑ Sometimes<br>❑ Often<br>❑ Always
Does your department/organization have appropriate job classifications, including position descriptions and pay scales for informatics positions? (If more than one is true, please select the option with the highest number)	❑ 0 - We have not attempted to adopt such informatics job classifications.<br>❑ 1 - We have made initial, isolated ad hoc efforts to adopt such informatics job classifications.<br>❑ 2 - We have made sustained attempts to adopt such informatics job classifications.<br>❑ 3 - We have adopted such informatics job classifications.<br>❑ 4 - We have adopted such informatics job classifications and established a method to evaluate implementation.

	❑ 5 - We use evaluation findings to improve how it recruits and retains informatics professionals.

Topic	Objectives
Intro to Public Health Informatics	▪ Explain the historical perspective of informatics and its relationship to the science of public health.<br>▪ Define terminology and vocabulary in public health informatics.<br>▪ Compare and contrast how public health information systems are evaluated at federal, state, and local public health agencies.<br>▪ Examine data standards and data analysis tools used in public health informatics to promote interoperability.<br>▪ Identify current and future challenges to the field of public health informatics.<br>▪ Describe the role of a Geographic Information System (GIS) in public health.
Epidemiology & Public Health Informatics	▪ Describe epidemiologic methods and research designs<br>▪ Explain incidence and prevalence rates<br>▪ Discuss the importance of understanding the denominator in any epidemiological study<br>▪ List several data sources<br>▪ Describe important issues in data management<br>▪ Provide an overview of information systems currently used in epidemiology<br>▪ Identify opportunities for more effective epidemiology investigation and disease prevention opportunities through implementation of informatic practices<br>▪ Describe challenges and opportunities presented by integration of information systems for epidemiology and disease prevention.
Health Data Science	▪ Abstract a business need for data analysis and define appropriate computational problem<br>▪ Design and analysis (time complexity) of simple algorithms<br>▪ List basic data structures and their characteristics, applications in biomedicine<br>▪ Retrieve biomedical data from multiple sources formats – specifically flat files (text), tabular data (CSV), structured data (JSON, XML)<br>▪ Implement Python programs to load data and apply basic data wrangling to structure output.
Privacy and Security	▪ Describe the standards of the HIPAA privacy rule<br>▪ Explain who is covered by the privacy rule and what information is protected<br>▪ Identify public health uses of data that may be exempt from the Privacy Rule.<br>▪ Critique privacy breach scenarios and identify the disclosure<br>▪ Distinguish between technical and non-technical safeguards<br>▪ Develop a cyber-related security incident response
Health Equity	▪ Discuss the evolution of health equity and health disparities as subjects of study and public health action.<br>▪ Identify the multiple dimensions of health disparities as described in Healthy People 2030 (US Dept of Health and Human Services, Office of Disease Prevention and Health Promotion; data-driven national objectives to improve health and be able to describe and critique approaches for studying the Social Determinants of Health.<br>▪ Discuss and analyze why the socioecological framework is critical for addressing health equity and reducing health disparities.<br>▪ Compare and contrast ethnic and racial influences on specific health inequities/health disparities in the U.S. population.<br>▪ Describe how the selection of reference groups can affect the measurement of health disparities and analyze, interpret, and present health equity data in tabular and graphic form.
Public Health Analytics	▪ Identify and define key terms and concepts associated with data analytics and public health data.<br>▪ Identify primary and secondary data sources of relevance to public health.<br>▪ Understand and contrast the functionality, advantages, and limitations of descriptive, predictive, prescriptive, and inferential analytical approaches.<br>▪ Identify public domain, census data, and associated resources to assess a public health problem.<br>▪ Explore the use of software solutions (R/Python, Commercial Off the Shelf) to manage and visualize public health datasets.
Surveillance in Public Health	▪ Define public health surveillance<br>▪ Describe the uses of a public health surveillance system<br>▪ Compare active and passive public health surveillance

	▪ Identify informatics tools used in active and passive public health surveillance<br>▪ Identify sources of data commonly used for public health surveillance<br>▪ Describe the public health surveillance process
Public Health Reporting	▪ Describe the history and importance of public health reporting of communicable and noncommunicable disease<br>▪ Differentiate between individual level and aggregate level data to be reported<br>▪ Differentiate between PHI vs. Non-PHI data<br>▪ Identify the need for secure level transmission and different codes for reporting (HL7 Vs FTP) and choose the appropriate level of security<br>▪ Identify the city county state and federal health agencies needed to report to<br>▪ Contrast reporting with surveillance
Semantic Interoperability	▪ Describe the difference between interoperability and semantic interoperability<br>▪ Explain why semantic interoperability is necessary for public health.<br>▪ Utilize HL7 FHIR in a given use case.<br>▪ Compare and contrast the uses of various health-related standards and select the best one for a given scenario.
Public Policy	▪ Discuss the history of the health information technology infrastructure and landmark legislation and their impact on public and population health<br>▪ Analyze the different stakeholder perspectives shaping health informatics policy<br>▪ Examine current issues relevant to health informatics policy, with Particular focus on recent health challenges<br>▪ Discuss the role of health informatics policies in influencing health disparities and inequities<br>▪ Develop a health informatics policy analysis
Multi-stream Data Management	▪ Define and describe multi-stream data in public health<br>▪ Describe and illustrate the architecture for managing multi-stream data<br>▪ Utilize data science methods to analyze multi-stream data<br>▪ Prepare multi-stream data for predictive analysis<br>▪ Apply multi-stream data for real-time public health dashboards
Social Media Listening	▪ Describe the influence of social media in the dissemination of health and safety information to create a more adaptive and effective public health response<br>▪ Explore the use of social media programmatic approaches to improve community/population health<br>▪ Investigate tools, techniques, and measures for public health social listening initiatives<br>▪ Describe best practices and tools for social media listening, network analysis and visualization for public health professionals<br>▪ Recognize challenges regarding privacy and security measures behind using social media platforms<br>▪ Identify critical challenges and opportunities for advancing social media listening for public health
Health Literacy	▪ Define health literacy and describe the role health literacy plays in meeting core public health services and health equity<br>▪ Identify who is affected by public health literacy and the consequences of limited public health literacy<br>▪ Identify health outcomes among people with low health literacy<br>▪ Describe why people, regardless of literacy skills, may fail to understand health information<br>▪ Recognize public health data, information and knowledge and the role played by misinformation and disinformation and how it can affect public health interventions<br>▪ Describe health literacy approaches or interventions that could be conducted to combat misinformation and disinformation
Racism & Bias in Data Use	▪ Define racism, implicit bias, explicit bias, structural racism, and antiracism<br>▪ Describe the historical origins of race in data science and informatics<br>▪ Explain how data and bias in machine learning can encode systemic racism<br>▪ Learn how to mitigate bias in data science processes<br>▪ Learn how to visualize all the real humans behind the aggregations<br>▪ Outline the essential role of data scientists in tackling racism and discrimination
Bias in Machine Learning and Artificial Intelligence	▪ Explain how explicit and implicit human biases can contribute to data biases<br>▪ Explain how the way in which data is sampled can introduce data biases even if there are no human biases in the data<br>▪ List methods to minimize the effects of bias in ML algorithms<br>▪ Describe multi-level approval for minimizing biases

Key Informant Interview Guide

INTRODUCTION

Good morning/afternoon, my name is _________ with the UT Health Science Center School of Public Health. We would like to thank you for completing the online survey and agreeing to take part in this interview. We provided a letter of information with additional information on our study, which supports the Gaining Equity in Training for Public Health Informatics and Technology (GET PHIT) Initiative. Our goal is to identify current needs relating to the public health informatics infrastructure and workforce in Texas. Do you have any questions pertaining to the initiative or study? Are you okay with me recording this interview? [If Yes] Once I begin the recording, I will ask you to confirm whether you agree to take part in the study and whether you agree to be recorded during this interview. After confirming, I will begin the interview.

Begin Recording
This is ______________ conducting interview #___ with participant #___. The date is _______.
Can you please state if you agree to take part in this study? Do you agree to be recorded during this interview? Thank you.

Before beginning our questions, I would like to share the definition of public health informatics.
The Centers for Disease Control and Prevention (CDC) defines public health informatics as the systematic application of information, computer science, and technology to public health practice, research, and learning. The application of informatics in public health includes the process by which raw data turns into digestible information to enable effective monitoring and surveillance and ensuring that practitioners have access to timely and accurate data. (https://phii.org/how-we-do-it/defining-public-health-informatics/)

QUESTIONS

1. How is informatics and the management of public health data part of the everyday job for staff at your department/organization?

Data Collection/Management/Sharing Practices

2. What gaps do you see in data sharing services?

3. What strategies have been implemented to improve data management/sharing internally and externally?

4. How does your department/organization consider equity in the distribution of data-specific information internally and externally?

5. Does your department/organization collect data (quantitative and qualitative) that showcase positive health measures in your community? (ex. civic engagement, prosocial behaviors, resilience, etc.) (Probing: how is the data collected?)

Informatics-Related Training

6. Describe your current training processes for informatics related positions? (Probing: Which positions are required to complete training? Do you cross-train staff from other departments?)

7. Is there a process for requesting informatics-related support across departments?

8. What are primary barriers to incorporating informatics-related training for all staff?

9. What resources are available to support informatics-related training and activities? (Probing: what financial support or % of your budget is allocated to this?)

<u>Workforce Development</u>

10. What type of education/certifications/experiences/skill sets would you like to see in future employees and interns?
